# Risk and Protective Factors for the Development of Career Resources: Results of the “Empower Peers 4 Careers” Intervention Study

**DOI:** 10.3390/bs16101808

**Published:** 2026-10-02

**Authors:** Claudia Schellenberg, Marlene Schulze, Patrizia Röösli, Annette Krauss

**Affiliations:** Institute for Educational Support for Behaviour, Social-Emotional, and Psychomotor Development (IVE), University of Teacher Education in Special Needs (HfH), 8050 Zurich, Switzerland; marlene.schulze@hfh.ch (M.S.); patrizia.roeoesli@hfh.ch (P.R.); annette.krauss@hfh.ch (A.K.)

**Keywords:** Positive Peer Culture, career resources, school-to-work transition, peer support, psychosocial difficulties, lower secondary education

## Abstract

The transition from school to work represents a key developmental challenge during adolescence. Career resources—personal, social, and identity-related factors that support career orientation and preparation—are crucial for navigating this transition. From a resilience perspective, the question arises under which risk and protective conditions such resources develop, are maintained, or are jeopardized. This study examines the relationship between psychosocial difficulties, a career-orientation-focused Positive Peer Culture (PPC) intervention, and the development of career resources. The study used a quasi-experimental pre-post design with an intervention and a control group. The sample consisted of 234 lower secondary school students from regular and special education schools who participated at both measurement points. Career resources were assessed with the Career Resources Questionnaire, and psychosocial difficulties with the Strengths and Difficulties Questionnaire. Linear regression analyses were conducted while controlling for baseline values and relevant sociodemographic variables. Exploratory subscale analyses and a process evaluation of the PPC group meetings were also conducted. The results showed no significant overall effect of the PPC intervention on career resources. However, psychosocial difficulties at baseline were a significant negative predictor of career resources in ninth grade. At the subscale level, an association with “Importance of Work in Life,” reflecting a motivational and value-related aspect of career development, emerged at the unadjusted level; however, this association did not remain statistically significant after correction for multiple testing and should therefore be considered exploratory. The process evaluation indicated predominantly positive group experiences, particularly collaborative problem-solving and mutual support, but also challenges regarding participation and implementation within the school schedule. The findings highlight the relevance of psychosocial difficulties for career development and suggest that the role of peer-based social support in career preparation warrants further investigation.

## 1. Introduction

The transition from compulsory schooling to general or vocational upper secondary education represents one of many important developmental tasks for adolescents during adolescence (Eschenbeck & Knauf, 2018). This developmental task can be considered particularly significant, as key decisions regarding future educational and career paths are made during this phase, and from a life-course perspective, one’s career plays a crucial role in determining social, health, and psychological circumstances (Dragano & Siegrist, 2009). However, not all adolescents have the same prerequisites for coping with these demands; a large proportion of 15- to 24-year-olds (16.4% of men and 29% of women) report experiencing moderate to severe psychological distress (Schweizerisches Gesundheitsobservatorium, 2025). Such psychosocial difficulties pose potential barriers to meeting developmental demands, as young people may resort to inappropriate coping strategies and exhibit lower academic performance, which is associated with a lower chance of graduating from school (Eschenbeck & Knauf, 2018; Plenty et al., 2021).

From a resilience perspective, particular importance is attached to promoting protective factors. Resilience describes the ability to successfully navigate developmental tasks despite stressful conditions. Supportive social relationships, as well as opportunities to develop a sense of competence and self-efficacy, are considered key protective factors in this context. Therefore, schools provide an important setting for supporting young people in their development (Steinebach et al., 2025). In particular, peer relationships represent a key resource in the school context, as they shape social learning processes, the sense of belonging, and the ability to overcome challenges. This is where the Empower Peers 4 Careers project comes in, which is based on the Positive Peer Culture (PPC) approach (Steinebach & Steinebach, 2013). It aims to specifically integrate peer relationships into career guidance instruction and to utilize them as a social resource for professional development.

### 1.1. Career Resources in the Transition from School to Work

Starting in eighth grade, adolescents face the challenge of exploring career options, discovering their own abilities, interests, visions for the future, and aspirations, making decisions, dealing with any uncertainties that may arise, and preparing themselves accordingly for the transition to the workforce. How young people deal with and cope with such demands depends largely on what resources and coping strategies are available to them (Eschenbeck & Knauf, 2018).

The transition from school to work can therefore be understood as a developmental challenge. Successfully navigating this transition depends on the interplay between general stressors, psychosocial difficulties, risk factors, protective factors, and available resources. However, developmental challenges are not automatically equivalent to stressors. Rather, stressors and risk factors may impair young people’s ability to mobilize available resources, whereas protective factors can support adaptation and strengthen resilience. In contrast, protective factors can promote the development or activation of resources and thus facilitate the positive management of career development tasks (Bengel et al., 2009). Accordingly, what matters is not solely the demands young people face, but rather the resources they have at their disposal to address and successfully manage these demands. Resources can generally be understood as prerequisites that have positive effects on development (Bengel et al., 2009).

For career development, this resource-oriented perspective can be specified using Hirschi’s career resources model (Hirschi, 2012). Career resources describe the prerequisites that can support young people in coping with career-related demands. Bengel et al. (2009) synthesize the findings on personal and social resources, while Hirschi (2012) also describes these in essence, but in a more nuanced way and in relation to necessary vocational competencies. The Career Resources Model distinguishes four resource areas: “human capital resources,” “social resources,” “psychological resources,” and “identity resources” (Hirschi, 2012, p. 376). These areas refer to different prerequisites for career development. While human capital resources primarily encompass knowledge, competencies, and qualification-related foundations, social resources relate to supportive relationships, contacts, and networks. Psychological resources describe psychological prerequisites that can support career-related actions and decision-making, such as confidence, optimism, or self-efficacy. Career identity resources focus on how individuals perceive themselves in relation to work, career goals, values, and the significance of career activities.

For adolescents in career preparation or during the transition from school to work, this resource-oriented approach was adopted and made measurable through a corresponding questionnaire. The Career Resources Questionnaire—Adolescent Version (CRQ-A; Marciniak et al., 2021) builds on Hirschi’s Career Resources Model (Hirschi, 2012) and is based on a conceptual adaptation of the Career Resources Questionnaire—originally developed for adults—to adolescents in a school setting. This is significant because, in relation to this developmental task, adolescents cannot yet be assessed in terms of occupational success in the narrow sense. Rather, they must be considered in terms of their preparation for working life and the resources that are relevant to this process. The CRQ-A therefore measures the key aspects of career preparation—that is, the attitudes, behaviors, and competencies that adolescents need to cope with career tasks, transitions, and challenges (Marciniak et al., 2021).

### 1.2. Factors Influencing Career Resources

This raises the question of the conditions under which the career-related resources presented here develop, are sustained, or are put at risk. As described earlier, from a resilience perspective, both protective and risk factors are significant for coping with career development demands (Bengel et al., 2009). While protective factors can support the development or activation of resources, risk factors increase the chance of unfavorable developmental trajectories by shifting the balance between stressors and available resources in an unfavorable direction.

This paper examines two selected factors influencing the development of career resources. First, psychosocial difficulties are measured using the Total Difficulties Score of the Strengths and Difficulties Questionnaire (SDQ) (Goodman, 2001). This is understood as an individual risk factor that reflects the component of psychosocial difficulties at the psychological level and thus represents a counterpart to supportive psychological resources. Second, the career-preparation-oriented Positive Peer Culture Intervention (Steinebach et al., 2018) is examined as a social-contextual support condition designed to promote the activation of social resources in the school setting, acting as a potential protective factor.

#### 1.2.1. Psychosocial Difficulties as an Individual Risk Factor

During adolescence, the majority of the adolescent succeed in coping with the central and various developmental demands. Nevertheless, this development does not proceed equally effortlessly for everyone. A significant proportion shows signs of psychological or psychosocial stress. In the BELLA Study, a study on the mental health of children and adolescents in Germany, 21.9% of respondents showed signs of mental health issues (Ravens-Sieberer et al., 2007). Data from Switzerland also highlight the significance of mental health challenges during adolescence. 18% of the population aged 15 and older experience moderate or severe psychological distress; in the 15- to 24-year-old age group, the proportion was 16.4% among men and 29.0% among women (Schweizerisches Gesundheitsobservatorium, 2025).

This illustrates that many children—and, of particular interest for this article, adolescents—are coping with psychosocial difficulties while navigating precisely those critical developmental challenges related to school and career.

In this article, psychosocial difficulties are understood as emotional, behavioral, and social difficulties that can impair adolescents’ experiences and behavior. Based on the classification along a behavioral dimension, these behavioral difficulties can be described as a set of specific problems in the areas of behavior and experience that frequently occur together and form dimensions or factors (Cullinan, 2004). A distinction is made between internalizing and externalizing behavior. Noticeable internalizing behaviors tend to be inwardly directed, for example, in the form of anxiety, depressed mood, or social withdrawal. Noticeable externalizing difficulties, on the other hand, tend to manifest through outwardly directed behavior, for example, in the form of aggression, opposition, or impulsivity, as well as hyperactivity and attention problems.

For the purposes of this article, it is important to note that such psychosocial difficulties are not merely understood as health problems but are rather linked to the process of coping with age-appropriate developmental challenges. For example, in their article Eschenbeck and Knauf (2018), make a very convincing case that psychological stress during adolescence can present potential barriers to coping with developmental demands and can lead to inappropriate coping strategies, or may even arise as a result of unsuccessful coping. Consequently, psychosocial difficulties can make the transition from school to work particularly difficult for these adolescents. Specifically, psychosocial difficulties can have negative effects on career preparation. Internalizing and externalizing problems are associated with lower academic performance and a lower chance of completing school (Plenty et al., 2021). Psychosocial difficulties can thus impair precisely those conditions that young people need in order to explore career options, reflect on their own abilities and interests, prepare for decisions, and make use of support. Against this backdrop, psychosocial difficulties are understood in this article as an individual risk factor that can adversely influence the development and change in career resources.

#### 1.2.2. Positive Peer Culture as a Social-Contextual Protective Factor

While psychosocial difficulties are understood as a risk factor related to individual circumstances, the Positive Peer Culture Intervention is designed to address the social resources aspect of the Career Resources Model. According to Bengel et al. (2009) and Hirschi’s model (Hirschi, 2012), social (career) resources lie outside the individual and include social relationships (e.g., family members and peers) and networks (e.g., mentors) that can be used to meet developmental needs. Relationships with peers represent a form of social resources that is particularly significant during adolescence, as they become increasingly relevant in this life phase as a context for orientation, exchange, and support (Steinebach & Steinebach, 2013).

The Positive Peer Culture (PPC) approach, originally developed by Vorrath and Brendtro (1974) and further developed within the framework of Peer Group Counseling, addresses precisely this need. In this way, adolescents become active supporters of one another and, in structured group discussions, bring their own challenges to the table, select topics that concern them, resolve problems together, and develop solutions. Adults (e.g., teachers, coaches) facilitate this process, ensuring that responsibility for addressing the content remains consciously with the young people themselves (Steinebach et al., 2018). Schellenberg et al. (2022) utilized this approach and adapted it to the context of career preparation in the Empower Peers 4 Careers project. The PPC group discussions are integrated into regular career preparation classes and focus on challenges related to career orientation, the development of realistic career aspirations, and preparation for vocational training and employment.

This is particularly important for the development of career resources, as peers can serve as role models, discussion partners, and sources of social support during the transition from school to work. For career-related development needs, for example, they can do so by sharing their career aspirations, experiences, and uncertainties with one another. It is precisely in this process that peer groups can create a space where they can compare their own perspectives with feedback from others, reflect on possible courses of action, and support one another in implementing planned steps (Schellenberg et al., 2022). In this sense, the adapted and implemented PPC approach should be understood as a potential social-contextual protective factor. This is intended to make social resources available within the school context. The intervention aims to facilitate mutual support among peers, strengthen a sense of belonging and the assumption of responsibility, and make social resources available within the school context. Since PPC primarily addresses social and reflective processes, potential effects on career resources are particularly plausible where career orientation is linked to exchange, support, attribution of meaning, and goal clarification.

### 1.3. Research Questions

Against this backdrop, the present study examines the extent to which selected risk and protective factors are associated with the level and development of career resources among eighth- and ninth-grade students. The focus is on psychosocial difficulties as an individual risk factor, as well as the Positive Peer Culture (PPC) intervention adapted for career preparation, which serves as a social-contextual support condition and potential protective factor. Additionally, the study examines how the intervention was implemented in the school setting and evaluated by the participating youth and the facilitators.

This gives rise to the following research questions respectively hypotheses:

**H1.**  *Does the level or change in career resources between the 8th and 9th school years differ between adolescents in the PPC intervention group and those in the control group?*

(H1) Adolescents in the PPC intervention group show more favourable changes in career resources between eighth and ninth grade than adolescents in the control group.

**H2.**  *To what extent are psychosocial difficulties associated with career resources in 9th grade, after controlling for baseline career resources and group membership?*

(H2) Higher levels of psychosocial difficulties are associated with lower levels of career resources in ninth grade, after controlling for baseline career resources and intervention group membership.

In addition, the study explores whether these associations vary across individual subdimensions of career resources.

**H3.**  *To what extent are PPC participation and psychosocial difficulties associated with individual subdimensions of career resources?*

Finally, to contextualize the intervention findings, an accompanying process evaluation addresses the following research question:

**H4.**  *How is the intervention’s implementation process evaluated in terms of acceptance among the adolescents and facilitators, as well as perceived benefits and group processes?*

## 2. Materials and Methods

### 2.1. Sample

The original baseline sample comprised 376 eighth-grade students from mainstream and special education schools at the lower secondary level who participated in the Empower Peers 4 Careers project. At baseline (T1), participants were enrolled in 33 eighth-grade classes (21 intervention and 12 control classes), with a follow-up assessment conducted in ninth grade (T2). The longitudinal sample comprised 234 adolescents from 29 classes (19 intervention and 10 control classes). Of the 376 adolescents who participated at T1, 234 (62.2%) also participated at T2 and were included in the longitudinal sample, whereas 142 (37.8%) were lost to follow-up.

An attrition analysis comparing retained participants with those lost to follow-up revealed differences in intervention versus control group membership (χ^2^(1) = 7.72, *p* = 0.005), school type (χ^2^(1) = 45.03, *p* < 0.001), gender (χ^2^(1) = 6.14, *p* = 0.013), and baseline age (*p* = 0.031). Retained participants were more frequently from the intervention group and mainstream schools, included a higher proportion of female students, and were slightly younger. No statistically significant baseline differences were observed in career resources (*p* = 0.818) or psychosocial difficulties (*p* = 0.067). These findings indicate that selective attrition may have affected the composition of the longitudinal sample.

The students attended mainstream schools at various achievement levels as well as special education schools for adolescents with socio-emotional difficulties. Thus, the longitudinal sample reflects different school settings relevant to examining developmental demands during the transition from school to work.

Table 1 presents the sociodemographic and baseline characteristics of the longitudinal sample at T1. The sample comprised 234 adolescents, of whom 151 (64.5%) were in the intervention group and 83 (35.5%) in the control group. The mean age of the participants was 14.23 years (*SD* = 0.62), 46.2% were female, and the majority attended a mainstream school (88.9%). Descriptively, participants in the intervention group were slightly older than those in the control group (*M* = 14.33, *SD* = 0.64 vs. *M* = 14.06, *SD* = 0.53). The proportion of female participants was similar across groups (45.7% in the intervention group vs. 47.0% in the control group), whereas a higher proportion of participants in the intervention group attended a mainstream school (94.7% vs. 78.3%). At baseline, there were no statistically significant differences between the intervention and control groups in CRQ or SDQ scores.

For the process evaluation, facilitators were also surveyed via an online questionnaire regarding the implementation and assessment of the PPC meetings. The sample comprised eleven facilitators. Six of them worked at a special education school and five at a mainstream school. The group included school-based special education teachers (*N* = 4), classroom teachers (*N* = 2), professionals in the field of social pedagogy (*N* = 3), and individuals in leadership roles (*N* = 2).

### 2.2. Study Design

The study follows a quasi-experimental pre-post design with an intervention and a control group. Data were collected at two time points: T1 (baseline) before the start of the intervention and T2 (posttest) after the intervention was completed. The intervention was conducted over a period of up to two school years. During this time, the adolescents in the intervention group regularly participated in PPC group meetings, while the control group received regular career preparation instruction without PPC. This design allows the analysis of changes over time as well as comparisons between the intervention and control groups while controlling for baseline values. Group allocation was non-randomized, as participating schools/classes could decide whether to implement PPC or participate as a control class without the intervention. Thus, self-selection into study conditions cannot be ruled out.

### 2.3. Intervention

The intervention is based on the Positive Peer Culture (PPC) approach and was integrated into regular classes, particularly in the context of career preparation. The main parts of the intervention are structured group discussions (peer group counseling), in which adolescents bring up their own problems and challenges and jointly develop solutions. The discussions follow a standardized eight-phase process, including problem identification, exploration, perspective-taking, and the development of action options (Schellenberg et al., 2022).

The role of the adult facilitators is primarily to facilitate the process by taking on an observational role during the peer group sessions and, for example, intervening if (discussion) rules are violated. In addition, they prepare a summary at the end and provide feedback on their observations. Responsibility for the content and course of the discussions is deliberately left to the young people. Prior to the program, the facilitators were prepared for their role in the intervention during a two-day training session at the University of Teacher Education in Special Needs (HfH), which the vast majority attended in full. In addition, up to four online supervision sessions were available, which were utilized to varying degrees; most facilitators participated in two to three sessions.

The PPC sessions took place primarily on a weekly or biweekly basis, lasting 45 min each. The planned intervention period extended over up to two school years. However, the actual duration varied across classes, with implementation in some settings lasting only one school year or being discontinued earlier. Accordingly, the interval between T1 and T2 was not identical across all participants. The number of PPC sessions conducted also varied across classes: 49 adolescents (32.5%) participated in fewer than eleven group sessions, while 47 adolescents (31.1%) attended eleven or more sessions. Information on the number of sessions was missing for 55 adolescents (36.4%). Due to these differences, the number of sessions conducted was taken into account as a measure of intervention intensity in supplementary analyses.

### 2.4. Survey Instruments

Quantitative data on the adolescents were collected using standardized paper-and-pencil questionnaires administered in the classrooms at both measurement time points. Psychological difficulties were assessed using the Strengths and Difficulties Questionnaire (SDQ) (Goodman, 2001). For the present analysis, the total difficulties score was used. This score is derived from the four problem scales: emotional problems, conduct problems, hyperactivity or attention problems, and peer problems. The prosocial behavior scale is not included in the total difficulties score. Higher scores indicate greater psychosocial stress. Internal consistency was evaluated using Cronbach’s alpha (α) and McDonald’s omega (ω). For the SDQ (20 items), the internal consistency of the total problem score was α = 0.75 and ω = 0.72 at T1.

Key career-related resources were assessed using the Career Resources Questionnaire (CRQ) (Marciniak et al., 2021) twice—at the beginning and at the end of the intervention. For the total career resources scale (CRQ; 16 items), the internal consistency was α = 0.75 and ω = 0.70 at T1 and α = 0.76 and ω = 0.71 at T2. The CRQ included items from eight subdimensions: The “Importance of Work in Life” subscale (2 items) showed comparatively low internal consistency at T2 (α = 0.53), which should be interpreted in light of the small number of items. The inter-item correlation was moderate (r = 0.36). The other subscales showed internal consistency coefficients ranging from α = 0.64 to α = 0.87 across both measurement time points. In addition, the adolescents in the intervention group were asked questions about the implementation and their perceptions of the PPC meetings. The survey measured participation in the meetings, general evaluation of the sessions, perceived well-being within the group, active participation, collaborative problem-solving, and perceived benefits of the discussions.

The quantitative survey of the facilitators was conducted using an online questionnaire created with LimeSurvey. It included custom-developed items assessing the intervention’s fidelity to the original design, facilitator behavior, and an evaluation of the method in terms of its implementation and its perceived impact on the adolescents.

### 2.5. Data Analysis

Statistical analyses were performed using the IBM SPSS Statistics software, version 29.0.2.0 (IBM Corp., 2023) to examine changes in the dependent variables as well as differences between the intervention and control groups. First, descriptive analyses were conducted to characterize the sample and the process variables.

Multiple linear regression analyses were conducted to examine predictors of career resources. The values of the respective variables at the second measurement point (T2) served as dependent variables. In the regression analyses, the respective baseline value (T1), group membership (participation in PPC: yes/no), psychosocial difficulties as measured by the Strengths and Difficulties Questionnaire (SDQ), and the covariates age, gender, and school type (regular or special education school) were simultaneously included as predictors. This approach corresponds to a pretest–posttest design with covariate control and allows for the examination of intervention effects while accounting for individual baseline differences and psychosocial difficulties at baseline. To assess the robustness of the main findings to within-class dependence, a supplementary linear mixed-effects model was estimated with a random intercept for school class, using the same predictors and analytical sample (*N* = 225) as in the main regression.

In addition, exploratory regression analyses were conducted at the subscale level of the Career Resources Questionnaire (CRQ). In each case, the corresponding subscale scores at the second measurement time point were used as dependent variables, and the respective baseline scores (T1) were controlled for, in order to identify differential effects of the intervention on individual dimensions of career resources. Because eight exploratory CRQ subscale models were examined, *p*-values were additionally adjusted for multiple testing using the Benjamini–Hochberg procedure to control the false discovery rate. Furthermore, within the intervention group, we examined whether the number of PPC sessions conducted was associated with the development of career resources. Missing values were handled using listwise deletion. The significance level was set at *p* < 0.05.

Of the 234 participants with data available at both T1 and T2, 225 (96.2%) were included in the regression analyses. Nine participants (3.8%) were excluded due to missing data on variables included in the regression models.

Table 2 presents the descriptive statistics for the CRQ total score and its subscales at T1 and T2. Overall career resources remained relatively stable, with a slight increase from T1 (*M* = 3.96, *SD* = 0.38) to T2 (*M* = 4.03, *SD* = 0.41). At the subscale level, general skills, clarity of career goals, self-reflection, and social support from peers increased descriptively, whereas importance of work in life and perceived social support from school and family decreased. The largest descriptive change was observed for social support at school.

## 3. Results

### 3.1. Predictors of Career Resources

Multiple linear regression analyses were conducted to examine predictors of career resources at T2. Baseline levels of career resources, group membership (PPC intervention vs. control group), psychosocial difficulties, age, gender, and school type (regular vs. special education school) were entered simultaneously as predictors (see Table 3). The model showed that participation in the PPC intervention was not significantly associated with career resources at T2 (β = −0.06, *p* = 0.34). In contrast, psychosocial difficulties at baseline were a significant negative predictor of career resources at T2, with a small standardized effect (β = −0.17, *p* = 0.01). This indicates that adolescents with higher levels of psychosocial difficulties at the start of the study showed lower levels of career resources in ninth grade. Baseline career resources also significantly predicted career resources at T2, with a moderate standardized effect (β = 0.30, *p* < 0.001). Age, gender, and school type were not significant predictors. Overall, the model explained 15% of the variance in career resources at T2 (*R*^2^ = 0.15, adj. *R*^2^ = 0.13), corresponding to a small-to-medium effect size (f^2^ = 0.18).

Since the number of PPC sessions varied across classes, a supplementary analysis was conducted within the intervention group to examine whether intervention intensity was associated with career resources at T2. Controlling for baseline career resources, age, gender, and school type, the number of PPC sessions was not significantly associated with career resources at T2 (β = 0.09, *p* = 0.414).

A supplementary linear mixed-effects model accounting for the nesting of students within school classes yielded a similar pattern of results. PPC participation remained non-significant (B = 0.045, *p* = 0.551), whereas psychosocial difficulties at baseline remained a significant negative predictor of career resources at T2 (B = −0.013, *p* = 0.012). The adjusted intraclass correlation coefficient was 0.074. Thus, the substantive conclusions of the main analysis remained unchanged after accounting for class-level dependence.

### 3.2. Subscale Analyses of Career Resources

To conduct an in-depth analysis of possible differential effects of the intervention, additional regression analyses were performed at the CRQ subscale level (see Table 4). The variables controlled for included baseline levels of career resources, age, gender, and school type (regular vs. special education school), as well as the psychosocial baseline situation (SDQ). In the exploratory subscale analyses, the smallest unadjusted *p*-value was observed for Importance of Work in Life (β = −0.16, *p* = 0.02). This subscale reflects the perceived personal importance and value attached to work and can therefore be considered a motivational and value-related aspect of career development. Descriptively, scores remained more stable in the intervention group than in the control group. However, this association did not remain statistically significant after adjustment for multiple testing (Benjamini–Hochberg; *p* = 0.16). This finding should therefore be regarded as preliminary and warrants further examination in future confirmatory studies. No clear evidence of PPC-related effects was observed for the remaining subscales.

In addition to the intervention effects reported in Table 4, psychosocial difficulties (SDQ) were included as covariates in all subscale models. Psychosocial difficulties significantly predicted the subscales “Importance of Work in Life” (β = −0.15, *p* = 0.025) and “Confidence” (β = −0.18, *p* = 0.008). Higher levels of psychosocial difficulties at baseline were thus associated with lower perceived importance of work and lower career-related confidence at T2. For the remaining CRQ subscales, psychosocial difficulties were not significant predictors.

### 3.3. Process Evaluation

In the following, as part of a multi-perspective process evaluation, the perspectives of the participating adolescents as well as the facilitators who conducted the intervention are presented. Descriptive results regarding the process evaluation of the PPC intervention from the adolescents’ perspective show, on the one hand, how much they enjoyed the sessions. Regarding the overall evaluation of the PPC sessions, 39.1% of participants (*N* = 59) selected a positive response category (“rather good” or “very good”), 28.5% (*N* = 43) selected the middle category (“partly”), and 32.5% (*N* = 49) selected a negative response category (“rather poor” or “not at all good”). The results further show that the sessions were generally rated positively overall (Table 5). Participants reported that they felt comfortable during the group discussions and actively participated in the discussions. In addition, it was noted that the group succeeded in jointly developing solutions to the problems raised. In contrast, the assessment of individual benefit was somewhat more reserved. The adolescents stated that the discussions helped them to a lesser extent in coping with their own problems.

In addition, the perspectives of the moderators are presented below. The descriptive results of the facilitator survey in Table 6 provide insight into the implementation of the intervention as well as its fidelity to the original design. A distinction is made between assessments regarding implementation by the students and those focusing on the facilitators’ actions.

The ratings regarding the students’ implementation of the PPC groups fall in the moderate to somewhat higher range: Particularly high scores are seen in maintaining confidentiality and in collaboratively developing solutions. Adherence to discussion rules is also rated comparatively positively. In contrast, the rating for the active participation of all group members is notably lower.

The facilitators’ assessments of their own actions and their facilitation role are mostly in the higher range. Particularly high scores are seen in adherence to key structural guidelines, i.e., following the prescribed procedure. Exercising influence without dominance and intervening with restraint are also rated positively. Assessments of their own restraint during discussions, however, tend to be lower.

In the overall assessment, it becomes clear that many facilitators rate highly the statement that young people can experience a sense of accomplishment through the method. By comparison, however, the assessment of the method’s ease of implementation in school settings is lower.

## 4. Discussion

### 4.1. Discussion of Key Findings

This study examined career start opportunities as measured by available career resources (Marciniak et al., 2021) in grades 8 through 9, as well as key factors influencing their development. On the one hand, the study examined the influence of the Empower Peers 4 Careers intervention as a context-specific social protective factor, assuming that the regular implementation of problem-centered peer groups (based on the “Positive Peer Culture” method, Steinebach et al., 2018) in career choice instruction is associated with a positive development of career resources. On the other hand, the adolescents’ initial psychosocial status (measured using the Strengths and Difficulties Questionnaire, SDQ) was also included as a risk factor, describing the presence of emotional difficulties, behavioral problems, concentration difficulties, and problems with peers. Based on the current body of research, it can be assumed that psychosocial risk factors may hinder the development of career-related resources or impair the transition from school to work (Eschenbeck & Knauf, 2018).

Contrary to expectations, the PPC intervention showed no significant effect on the overall career resources scale. Nor was the number of group sessions conducted associated with the development of career resources. The results thus suggest that participation in the PPC groups alone was not sufficient to bring about comprehensive changes in the adolescents’ career-related resources. This finding may seem surprising at first, as career resources are considered important prerequisites for coping with career development tasks (Marciniak et al., 2021). At the same time, the results are consistent with earlier findings from the project, which demonstrated positive effects of PPC primarily on social variables such as school integration (Krauss et al., 2026). Theoretically, it is also assumed that PPC primarily operates through social processes. At the heart of the approach are mutual support, taking on responsibility, and the experience of belonging within the peer group (Steinebach et al., 2018). Accordingly, PPC appears to strengthen social protective factors initially, while changes in individual developmental characteristics may only become apparent in the longer term.

At the subscale level, the exploratory analyses yielded the smallest unadjusted *p*-value for the dimension “Importance of Work in Life” (β = −0.16, *p* = 0.02). However, this association did not remain statistically significant after adjustment for multiple testing and should therefore be interpreted cautiously. Descriptively, the pattern was consistent with a more stable trajectory in the intervention group. This observation may nevertheless be of interest from a hypothesis-generating perspective, as the subscale reflects attitudes and value orientations toward work rather than specific career-related competencies. The PPC discussions provided opportunities for adolescents to reflect jointly on experiences, concerns, and future perspectives related to the transition from school to work. It is therefore conceivable that such exchange processes may be more closely related to motivational and value-related aspects of career development than to immediate changes in specific competencies.

A key finding of the study concerns the role of psychosocial difficulties. The SDQ total problem score proved to be a significant predictor of career resources at the second measurement point. Adolescents with lower levels of psychosocial difficulties at the start of the study later possessed higher levels of career resources. This finding supports theoretical considerations from resilience research. Developmental tasks such as career choice require not only information about occupations or specific job application skills, but also emotional stability, self-regulation skills, and social resources (Masten et al., 2021). Psychological difficulties can make it difficult to actively engage with one’s professional future and can impair the use of available support services. The subscale analyses further specify this pattern. Psychosocial difficulties were particularly associated with the dimensions “Importance of Work in Life” and “Confidence.” This suggests that psychosocial difficulties may be especially relevant for motivational and self-related aspects of career development. Adolescents experiencing psychosocial difficulties may find it more difficult to perceive work as personally meaningful and to develop confidence in their own career-related abilities (Hirschi, 2012; Marciniak et al., 2021).

The results thus highlight the close link between mental health and career development. The transition from school to the working world cannot be understood solely as an educational or career process; rather, it also represents a significant psychosocial developmental challenge. Interventions designed to support career choice should therefore take into account not only academic aspects but also the mental health and social support of young people. From a resilience perspective, PPC appears particularly interesting in this context. The concept aims to build social protective factors, promote the assumption of responsibility, and strengthen supportive relationships among young people. Such protective factors are considered key resources for coping with developmental tasks and could also support career development in the long term (Eschenbeck & Knauf, 2018). Future studies should therefore investigate whether the effect of PPC on career resources is partially mediated by changes in psychosocial difficulties or social protective factors.

The results of the process evaluation are consistent with the assumption that PPC operates through social processes. The adolescents reported generally positive group experiences, active involvement, and successful collaborative problem-solving. At the same time, the overall evaluation of the PPC sessions was mixed, with approximately one third of participants expressing a more critical view. This indicates that not all adolescents were equally motivated or engaged by the intervention. At the same time, they were more reserved in their assessment of the direct benefits for their own problem-solving. The facilitators also rated the intervention predominantly positively. In particular, they highlighted the opportunity to foster a sense of achievement, delegate responsibility to adolescents, and promote collaborative problem-solving. These findings underline the importance of facilitator training that supports both the active involvement of less participative students and a consistently non-directive facilitation style. At the same time, it became apparent that ensuring the active participation of all adolescents and the facilitators’ consistent restraint posed challenges. These findings align well with the theoretical foundations of Positive Peer Culture. This approach views development as a social process and emphasizes the importance of mutual support within the group (Steinebach et al., 2018). The positive evaluations of the group processes, as well as the perception of collaborative problem-solving, suggest that central elements of the PPC concept were successfully implemented. At the same time, the results show that implementation in everyday school life remains challenging and that designing sustainable peer processes requires time and appropriate framework conditions (Wilson & Lipsey, 2007).

### 4.2. Limitations and Outlook

The present results must be interpreted in light of several limitations. First, the study employs a quasi-experimental design, which limits the possibility of drawing causal conclusions. Although no significant baseline differences were observed in career resources and psychosocial difficulties and relevant baseline and sociodemographic variables were controlled for, unmeasured differences between the intervention and control groups cannot be ruled out because group allocation was non-randomized. Although a supplementary linear mixed-effects model accounting for class-level dependence yielded similar findings, the limited number of classes warrants caution when interpreting the precision of the estimates.

Second, the PPC groups were implemented under different school-based conditions. The number of sessions held varied considerably between classes, as did the organizational structure and the composition of the groups. Such heterogeneity can lead to so-called dilution effects, in which potential intervention effects are weakened. Although the number of sessions held was analyzed separately, no significant correlations with career resources were found. At the same time, no systematic data were available on the quality of implementation or on actual adherence to the program design. Differences in the design of the peer processes, youth participation, or facilitation practices could therefore not be examined in depth. However, based on previous research, it can be assumed that both the intensity and the quality of implementation are crucial for the effectiveness of PPC (Otto, 2016).

Third, the analyses are based primarily on self-reports from the adolescents. Particularly for constructs such as career resources, psychosocial difficulties, or group processes, subjective assessments may be influenced by social desirability or individual response patterns. In addition, individual subscales of the Career Resources Questionnaire exhibit comparatively low reliability, which limits the interpretation of the subscale findings. Given the exploratory nature of the eight subscale regressions and the multiple statistical tests conducted, these findings should be regarded as hypothesis-generating rather than confirmatory.

A further limitation concerns attrition between T1 and T2. Of the original baseline sample, 37.8% were lost to follow-up. Attrition differed by intervention group, school type, gender, and age. Although retained participants did not differ significantly from those lost to follow-up in baseline career resources or psychosocial difficulties, selective attrition may have affected the comparability of the groups and the generalizability of the findings.

A further limitation concerns the interpretation of PPC within a resilience framework. Although psychosocial difficulties were conceptualized as an individual risk factor and PPC as a potential social-contextual protective factor, the present analyses examined their associations with career resources as separate predictors. No interaction between psychosocial difficulties and intervention status was tested. Therefore, whether PPC buffers the negative association between psychosocial difficulties and career resources remains an open question for future research.

Despite these limitations, the study provides important insights into the relationships between peer support, mental health, and career development during adolescence. Of particular note is the finding that psychosocial difficulties are a significant predictor of career resources. This underscores the need to view career guidance not in isolation, but rather to link it more closely to issues of mental health and resilience.

For future research, it would be useful to examine the interactions between psychosocial difficulties and career development in even greater depth. Further questions could include whether adolescents with increased psychosocial difficulties initially require greater support in the areas of emotional and social stabilization before they can benefit from career-choice-related services. It would also be worth examining whether PPC can help adolescents with psychosocial difficulties in particular build protective factors such as a sense of belonging, social support, and self-efficacy, which may not impact career development processes until a later stage.

Furthermore, longer observation periods would be desirable. It is conceivable that the effects of PPC on career development processes may only become apparent after a time lag and, for example, emerge during the transition to vocational training or when facing initial career challenges. Future studies could therefore investigate whether the social protective factors developed through PPC contribute in the long term to a more successful transition from school to the working world.

## 5. Conclusions

The findings of this study suggest that Positive Peer Culture (PPC) should not primarily be understood as a direct intervention for increasing general career resources. Rather, the process evaluation suggests that PPC may contribute to favorable social conditions for developmental and learning processes by promoting mutual support, collaborative problem-solving, reflection, and a sense of belonging. This interpretation is consistent with previous findings from the project showing positive effects on school integration (Krauss et al., 2026). Although no significant overall effect on career resources was found, the exploratory subscale analyses yielded a tentative indication regarding the perceived significance of work, a motivational and value-related aspect of career development. This observation warrants further investigation.

A central finding is that psychosocial difficulties at baseline were negatively associated with later career resources. This highlights the importance of considering mental health and psychosocial functioning in career guidance. Adolescents experiencing psychosocial difficulties may need additional emotional and social support in order to engage with career-related demands and make use of available resources.

For school practice, the findings, together with previous research, point to the potential value of linking career guidance more closely to resilience-building, social support, and psychosocial prevention. Career choice programs may therefore benefit from being combined with approaches designed to promote socio-emotional skills, socio-emotional learning, or school psychological support services (Casale et al., 2014). Peer-based approaches such as PPC may provide a useful framework for strengthening social protective factors, particularly when they are embedded in a broader school culture of mutual support and implemented with sufficient time, preparation, and facilitation (Steinebach et al., 2018). Future research should further examine how social protective factors, psychosocial difficulties, and career development interact over time (Schellenberg et al., 2020).

## Figures and Tables

**Table 1:** Sociodemographic Characteristics of the Sample (T1).

Sample Characteristics	Total Sample	Intervention Group	Control Group
N	234	151	83
Age	14.23 (0.62)	14.33 (0.64)	14.06 (0.53)
Female	108 (46.2%)	69 (45.7%)	39 (47.0%)
Regular school	208 (88.9%)	143 (94.7%)	65 (78.3%)
CRQ T1	3.96 (0.38)	3.99 (0.37)	3.90 (0.39)
SDQ T1	12.99 (5.33)	13.26 (5.10)	12.49 (5.74)

Notes: *M* = mean; *SD* = standard deviation. Percentages refer to the respective sample or group.

**Table 2:** Descriptive statistics for the variables examined at T1 and T2.

Variable	T1		T2	
	*M*	*SD*	*M*	*SD*
Career Resources (CRQ): Total Scale	3.96	0.38	4.03	0.41
CRQ1: Importance of Work in Life	4.41	0.50	4.33	0.64
CRQ2: General Skills	3.85	0.66	4.03	0.57
CRQ3: Confidence	4.09	0.54	4.15	0.58
CRQ4: Clarity of Career Goals	4.00	1.00	4.09	0.86
CRQ5: Self-Reflection	3.96	0.70	4.11	0.67
CRQ6: Social Support at School	4.32	0.68	4.00	0.82
CRQ7: Social Support from Family	4.32	0.72	4.20	0.84
CRQ8: Social Support from Peers	3.19	0.99	3.34	1.00

Notes: Valid responses varied between *N* = 226 and *N* = 232 (T1) and *N* = 225–232 (T2) across items. *M* = mean; *SD* = standard deviation. All scales were measured on a five-point Likert scale (1 = low level, 5 = high level).

**Table 3:** Results of the multiple regression analysis predicting career resources (CRQ T2).

Predictor	B	SE	Beta	*p*
CRQ T1	0.32	0.07	0.30 **	<0.001
Participation PPC	−0.05	0.06	−0.06	0.34
SDQ T1	−0.01	0.01	−0.17 *	0.01
Age	−0.01	0.05	−0.02	0.79
School Type	0.09	0.09	−0.06	0.32
Sex	−0.05	0.05	0.07	0.33

Notes: *N* = 225. AV = Career resources at T2. CRQ = Career Resources Questionnaire. PPC = Positive Peer Culture, group membership: 1 = intervention group, 2 = control group, * *p* < 0.05, ** *p* < 0.01; *R*^2^ = 0.15, adj. *R*^2^ = 0.13.

**Table 4:** Effects of PPC participation on the subscales of career resources (CRQ): Exploratory subscale analyses.

Subscale	B	Beta	*p*
**Importance of Work in Life**	**−0.21**	**−0.16**	**0** **.02**
General skills	0.05	0.04	0.52
Confidence	−0.13	−0.11	0
Clarity about career goals	−0.18	−0.10	0.16
Self-Reflection	0.00	0.00	0.97
Support from school	0.05	0.03	0.66
Support from family	0.05	0.03	0.70
Support from friends	0.01	0.00	0.95

Notes: Group membership was coded as 1 = intervention group and 2 = control group. Valid cases varied between *N* = 211 and *N* = 222 across models. B = unstandardized regression coefficient; β = standardized regression coefficient. The table reports the regression coefficients for PPC participation in separate regression models for each CRQ subscale at T2. Models controlled for the corresponding baseline subscale score, psychosocial difficulties, age, gender, and school type. *p*-values are reported unadjusted. Using a Benjamini–Hochberg correction, none of the associations remained statistically significant.

**Table 5:** Descriptive results of the process evaluation of the PPC intervention from the adolescents’ perspective.

Variable	*M*	*SD*
Overall evaluation of PPC sessions	3.10	1.20
I felt comfortable during the group discussions	3.39	1.07
We were able to find solutions together	3.59	1.00
I actively participated in the discussions	3.43	1.05
The discussions helped me with my problems	2.81	1.16

Notes: Valid responses varied between *N* = 147 and *N* = 151 across items. *M* = mean; *SD* = standard deviation. Respondents rated the items on a five-point Likert scale (1 = strongly disagree/negative rating, 5 = strongly agree/positive rating).

**Table 6:** Selected results from the process evaluation of the PPC intervention from the facilitators’ perspective.

Area	Item	*M*	*SD*
Implementation by Students	The students are able to work together to develop solutions.	2.95	0.75
	The rules are followed.	2.60	0.75
	What is discussed remains confidential.	3.06	0.59
	All students in the group actively participate in the discussion.	1.68	1.37
Facilitators’ Actions	I follow the guidelines of the procedure as closely as possible.	3.11	0.58
	I can leave the responsibility for solving problems to the young people.	2.94	0.80
	I have influence without dominating the group.	3.06	0.68
	I intervene as little as possible, but as often as necessary.	3.27	0.66
	I find it easy to hold back during a conversation.	2.38	0.97
Evaluation of the Intervention	This method allows young people to experience a sense of accomplishment.	3.47	0.51
	The method can be easily implemented in the school setting.	2.23	1.09

Notes: *N* = 11. *M* = mean; *SD* = standard deviation. Ratings were given on a four-point scale (0 = never/strongly disagree to 4 = always/strongly agree). Higher values indicate greater agreement or more frequent implementation of the respective aspects.

## Data Availability

The data supporting the findings of this study are not publicly available due to privacy and ethical restrictions related to research with minors in a school context. Aggregated data may be made available from the corresponding author upon reasonable request.

## References

[B1-behavsci-16-01808] Bengel J., Meinders-Lücking F., Rottmann N. (2009). Schutzfaktoren bei kindern und jugendlichen: Stand der forschung zu psychosozialen schutzfaktoren für gesundheit.

[B2-behavsci-16-01808] Casale G., Hennemann T., Hövel D. (2014). Systematischer überblick über deutschsprachige schulbasierte maßnahmen zur prävention von verhaltensstörungen in der sekundarstufe I. Empirische Sonderpädagogik.

[B3-behavsci-16-01808] Cullinan D. (2004). Classification and definition of emotional and behavioral disorders. Handbook of research in emotional and behavioral disorders.

[B4-behavsci-16-01808] Dragano N., Siegrist J. (2009). Die lebenslaufperspektive gesundheitlicher ungleichheit: Konzepte und forschungsergebnisse. Gesundheitliche ungleichheit.

[B5-behavsci-16-01808] Eschenbeck H., Knauf R.-K., Lohaus A. (2018). Entwicklungsaufgaben und ihre bewältigung. Entwicklungspsychologie des jugendalters.

[B6-behavsci-16-01808] Goodman R. (2001). Psychometric properties of the strengths and difficulties questionnaire. Journal of the American Academy of Child & Adolescent Psychiatry.

[B7-behavsci-16-01808] Hirschi A. (2012). The career resources model: An integrative framework for career counsellors. British Journal of Guidance & Counselling.

[B8-behavsci-16-01808] IBM Corp (2023). IBM SPSS statistics for windows *(Version 29.0.2.0) [Computer software]*.

[B9-behavsci-16-01808] Krauss A., Schellenberg C., Röösli P. (2026). Positive peer culture zur stärkung schulischer integration. Ergebnisse eines präventionsprojekts auf sekundarstufe I. Prävention und Gesundheitsförderung.

[B10-behavsci-16-01808] Marciniak J., Hirschi A., Johnston C. S., Haenggli M. (2021). Measuring career preparedness among adolescents: Development and validation of the career resources questionnaire—Adolescent version. Journal of Career Assessment.

[B11-behavsci-16-01808] Masten A. S., Lucke C. M., Nelson K. M., Stallworthy I. C. (2021). Resilience in development and psychopathology: Multisystem perspectives. Annual Review of Clinical Psychology.

[B12-behavsci-16-01808] Otto A. (2016). Fachbeitrag: Peerkulturelle praktiken zwischen möglichkeitsraum und schulischer ordnung. Schülerperspektiven auf den arbeitsansatz positive peerkultur im schulischen handlungsraum. Zeitschrift Vierteljahresschrift für Heilpädagogik und ihre Nachbargebiete.

[B13-behavsci-16-01808] Plenty S., Magnusson C., Låftman S. B. (2021). Internalising and externalising problems during adolescence and the subsequent likelihood of being not in employment, education or training (NEET) among males and females: The mediating role of school performance. SSM—Population Health.

[B14-behavsci-16-01808] Ravens-Sieberer U., Wille N., Bettge S., Erhart M. (2007). Psychische gesundheit von kindern und jugendlichen in deutschland: Ergebnisse aus der BELLA-studie im kinder- und jugendgesundheitssurvey (KiGGS). Bundesgesundheitsblatt—Gesundheitsforschung—Gesundheitsschutz.

[B15-behavsci-16-01808] Schellenberg C., Krauss A., Pfiffner M., Georgi-Tscherry P. (2020). Inklusive didaktik und nachteilsausgleich an berufs fachschulen und gymnasien. Schweizerische Zeitschrift für Heilpädagogik.

[B16-behavsci-16-01808] Schellenberg C., Steinebach C., Krauss A. (2022). Empower peers 4 careers: Positive peer culture to prepare adolescents’ career choices. Frontiers in Psychology.

[B17-behavsci-16-01808] Schweizerisches Gesundheitsobservatorium (2025). Psychische gesundheit in der schweiz: Entwicklung, förderung, prävention und versorgung. Nationaler gesundheitsbericht 2025.

[B18-behavsci-16-01808] Steinebach C., Knafla I., Schär M., Avci-Werning M., Gawrilow C. (2025). Resilienz in der schule. Schulpsychologie.

[B19-behavsci-16-01808] Steinebach C., Schrenk A., Steinebach U., Brendtro L. K. (2018). Positive peer culture: Ein manual für starke gruppengespräche.

[B20-behavsci-16-01808] Steinebach C., Steinebach U., Steinebach C., Gharabaghi K. (2013). Gleichaltrige: Peers als ressource. Resilienzförderung im jugendalter.

[B21-behavsci-16-01808] Vorrath H. H., Brendtro L. K. (1974). Positive peer culture.

[B22-behavsci-16-01808] Wilson S. J., Lipsey M. W. (2007). School-based interventions for aggressive and disruptive behavior. American Journal of Preventive Medicine.

